# Beyond traditional metrics: A novel method for measuring mood instability in bipolar disorder

**DOI:** 10.21203/rs.3.rs-3880090/v1

**Published:** 2024-01-24

**Authors:** Sarah Sperry, Anastasia Yocum, Melvin McInnis

**Affiliations:** University of Michigan; University of Michigan; University of Michigan

## Abstract

**Background:**

Clinical care for bipolar disorder (BD) has a narrow focus on prevention and remission of episodes with pre/post treatment reductions in symptom severity as the ‘gold standard’ for outcomes in clinical trials and measurement-based care strategies. The study aim was to provide a novel method for measuring outcomes in BD that has clinical utility and can stratify individuals with BD based on mood instability.

**Methods:**

Participants were 603 with a BD (n=385), other or non-affective disorder (n=71), or no psychiatric history (n=147) enrolled in an intensive longitudinal cohort for at least 10 years that collects patient reported outcomes measures (PROMs) assessing depression, (hypo)mania, anxiety, and functioning every two months. Mood instability was calculated as the within-person variance of PROMs and stratified into low, moderate, and high thresholds, respectively.

**Outcomes:**

Individuals with BD had significantly higher mood instability index’s for depression, (hypo)mania, and anxiety compared to psychiatric comparisons (moderate effects, p’s<.001) and healthy controls (large effects, p’s<.001). A significantly greater proportion of individuals with BD fell into the moderate (depression: 52·8%; anxiety: 51·4%; (hypo)mania: 48·3%) and high instability thresholds (depression: 11·5%; anxiety: 9·1%; (hypo)mania: 10·8%) compared to psychiatric comparisons (moderate: 25·5 – 26·6%; high: 0% - 4·7%) and healthy controls (moderate: 2·9% - 17·1%; high: 0% - 1·4%). Being in the high or moderate instability threshold predicted worse health functioning (p’s < .00, small to large effects).

**Interpretation:**

Mood instability, as measured in commonly used PROMs, characterized the course of illness over time, correlated with functional outcomes, and significantly differentiated those with BD from healthy controls and psychiatric comparisons. Results suggest a paradigm shift in monitoring outcomes in BD, by measuring mood instability as a primary outcome index.

## 1. Introduction

Bipolar disorders (BD) are among the leading causes of disability worldwide due to early onset, high chronicity, and comorbidity rates (1). Premature mortality associated with BD equals or surpasses that of several common risk conditions, including smoking and cardiovascular disease (2). Despite this, accurate diagnosis is often delayed and, even when diagnosed properly, efficient treatment options remain stagnant (3). Traditional nosology and classification describe BD as relapsing disorders during which distinct episodes of mania, hypomania, and depression vacillate with remitted periods. These remitted periods have long distinguished BD from primary psychotic disorders or borderline personality disorder, and are based on a return to normal mood or “euthymia” in between episodes (4). However, the recent emphasis on intensive longitudinal designs in BD research has begun to paint a sobering picture of these patterns, challenging traditional nosology and de nitions of euthymia (5, 6).

Efforts to model the course of BD across both micro (hourly) and macro (monthly to yearly) timescales have proliferated over the past decade. Using methods such as ecological momentary assessment (EMA) and intensive longitudinal cohort designs, time series analysis, and mathematical modeling finds that individuals with BD, as well as those at risk for BD, experience significant instability (fluctuations and deviations away from one’s average state) in emotions and mood even outside the context of mood episodes (7–12)^[1]^. Importantly, mood instability is associated with important risk and outcomes. In one study, day-to-day mood instability predicted the development of bipolar but not unipolar disorders three years later (13). Furthermore, several studies have established that mood instability, outside the context of mood episodes, is associated with worse outcomes and poorer functioning (14–16).

Despite converging evidence that mood instability is a core phenotypic feature of BD, clinical care continues to have a narrow focus on prevention and remission of episodes. Symptom severity reduction is the ‘gold standard’ for outcomes in clinical trials of novel interventions; the ‘measurement-based’ care strategy is likewise motivated towards achieving a specific integer threshold on gold standard measures of mood. Success is typically defined by simple linear decreases in scores from two (pre-post treatment) to three time points (pre-treatment, mid-treatment, post-treatment). We, along with others (5, 17), argue that for substantial improvements in diagnosis, care, and treatment development, alternative ways to measure change in BD based on mood instability are needed.

In this study, a unique cohort of individuals from the Prechter Longitudinal Study of Bipolar Disorder (PLS-BD) (18, 19) was leveraged. The PLS-BD includes individuals with BD, a psychiatric comparison (PC) group with non-BD diagnoses, and individuals with no psychiatric history (HC) who completed mood measures every two months for a minimum of 10 years. Using this intensive longitudinal data, mood instability was characterized across diagnostic categories to determine if thresholds could be determined that stratify individuals with low, moderate, and high levels of instability. Furthermore, the hypothesis that being in a higher instability threshold would be associated with lower levels of functioning was tested. Instability thresholds are based on clinically relevant measures, widely administered across medical systems, and are a part of standard of care such as the Patient Health Questionnaire (PHQ-9), Altman Self-Rating Mania Scale (ASRM), and the Generalized Anxiety Scale (GAD-7). The GAD-7 was included as anxiety disorders are highly comorbid with BD, anxiety tends to fluctuate with depression in BD (20), and it provides generalizability beyond mood episode symptoms (mania, depression).

## 2. Methods

### 2·1 Study design and participants

Participants were drawn from the PLS-BD, an ongoing cohort study of BD that has been continuously gathering phenotypic and biological data over the naturalistic course of BD beginning in 2006. Participants are recruited via advertisements, psychiatric clinics, mental health centers, and community outreach events in Michigan. Participants are excluded if diagnosed with neurological disease or alcohol or substance use that would interfere with the ability to complete research (e.g., attending interviews intoxicated). The present study included 603 participants (Mean_Age_ = 39, SD_Age_= 14, 65·7% female, 78·6% White, 21·4% non-Caucasian, Mean_enrollment_ = 13 years, Range_enrollment_ = 10–17) who had completed at least 10 years of follow-up in the study. Participants in the BD group had a DSM-IV-TR diagnosis of BD I (n = 258), BD II (n = 80), BD Not Otherwise Specified (n = 30), or Schizoaffective BD (n = 17). Participants in the PC group had a DSM-IV-TR diagnosis of major depressive disorder (n = 20), non-affective diagnoses (n = 23), or other affective diagnoses (n = 28). The HC included individuals (n = 147) who had no psychiatric history and no first-degree relatives with BD. Diagnosis was assessed using the Diagnostic Interview for Genetic Studies, version 4 (21). A team of at least two doctoral-level psychologists or psychiatrists confirmed diagnosis using criteria from the DSM-IV-TR and all available medical history from electronic health records. Demographic and descriptive information regarding each group is presented in Supplemental Tables 1–4. Written informed consent was obtained from participants and all study procedures were approved by the Institutional Review Board at Michigan Medicine.

### 2·2 Choice of primary measure

The full longitudinal protocol and procedures of the PLS-BD are outlined elsewhere (19). We focus on measures related to the current investigation which include mood and functioning measures that are administered every two months for the duration of the study. Demographics including sex at birth, race, ethnicity, and age of onset are collected annually on the participants’ anniversary of enrollment while age was calculated as of November 1, 2023 the date at which data was extracted for analysis.

Self-reported depression symptoms over the past two weeks were measured using the gold-standard nine-item Patient Health Questionnaire (PHQ-9) (22). Items are answered on a Likert Scale from 0 (Not at all) to 3 (Nearly every day) with a scale score range from 0 to 27. Scores of 5 to 9 indicate mild depression, 10–14 moderate depression, 15–19 moderately severe depression, and 20–27 severe depression. Self-reported manic symptoms over the past two weeks were measured using the five-item Altman Self-Rating Mania Scale (ASRM) (23). Items are answered on a scale from 1 to 5 with scale scores ranging from 5 to 25. A score of 6 or higher indicates a concern for manic or hypomanic presentations. Self-reported anxiety symptoms were measured using the gold-standard seven-item Generalized Anxiety Disorder Scale (GAD-7) (24). Items are answered on a scale from 0 (Not at all) to 3 (Nearly every day). Scores range from 0 to 21 (0–4: minimal, 5–9: mild, 10–14: moderate, >= 15: severe anxiety). General health and functioning were measured using the short form of the SF-36, the SF-12 (25). The SF-12 is a widely used measure to assess quality of life in health-sciences. The SF-12 results in two normed T scores (mean = 50, SD = 10), the mental component summary (MCS) and physical component summary (PCS), with higher scores indicating better-than-average functioning. Internal consistency for all measures were good to excellent in the current sample (Chronbach’s a = 0·82 − 0·92).

### 2·3 Analytical Strategy

Rolling variance of the PHQ-9, ASRM, and GAD-7 was calculated for each participant using three window widths; 3, 6, and 12 corresponding to 3, 6, and 12 independent measures captured over 6-months, 1-year, and 2-years, respectively. Visual inspection with a LOESS (Locally Weighted Scatterplot Smoothing) line was completed along with calculation of the Mean Squared Error (MSE) and standard deviation (SD) to examine responsiveness of each window width. One-year of rolling variance was selected as it had the lowest MSE but highest SD across 20 random selected participants representing 12:4:4 ratio of BD:PC:HC. Raw rolling variances were z-scaled so that the distributions were similar and comparable across participants (continuous measure of instability). To identify categorical thresholds of low, moderate, and high instability based on rolling variances, independent of diagnostic group, ranked percentiles were created (n = 100). Thresholds were determined from ranked percentiles so that </= 60-%tile was considered “low”, 61–94-%tile was “moderate” and >/= 95-%tile was considered “high” instability for each measured scale. As such, each participant had a continuous index and categorical threshold of instability for each mood measure.

To account for multiple comparisons and reduce chances of type I error with a large number of within-person observations, we adjusted the alpha level to .001 across all models. To examine whether diagnostic groups differed in terms of continuous measures of instability, linear mixed-effects models were used in the *lmer* package in R. To examine pair-wise group differences, we completed post-hoc contrasts with Tukey correction for multiple comparisons. To test whether there were proportional differences in the low, moderate, and high thresholds by diagnosis, we ran pair-wise chi-square tests. To test whether thresholds predicted longitudinal functioning outcomes (mental and physical health functioning from the SF-12), over and above other important covariates (diagnosis, sex, race, age), we ran linear mixed-effect models robust to missingness. An example formula is provided below:

(1) $$SF12\_MCS_{ij}={\text{Y}}_{00}+{\text{Y}}_{01}{\left(\text{diagnosticgroup}\right)}_{j}+{\text{Y}}_{02}{\left(sex\right)}_{j}+{\text{Y}}_{03}{\left(\text{race}\right)}_{j}+Y_{04}{\left(age\right)}_{j}+{\text{Y}}_{10}{\left(\text{instabilitythreshold}\right)}_{ij}+U_{0j}+R_{ij}$$

SF12_MCS = mental health functioning. Diagnostic group (0 = HC), sex (0 = male), race (0 = White). represents a random intercept for each participant.

## 3. Results

Calculating instability based on the one-year rolling variance for each mood measure resulted in 20,854 observations for depression, 21,199 for mania, and 14,105 for anxiety[2]. Summary statistics for all variables are provided in Supplemental Tables 1–4 and zero-order spearman rho within- and between-person correlations are presented in Supplemental Figure 1.

### 3·1 ASRM Instability

**Figure 1:**
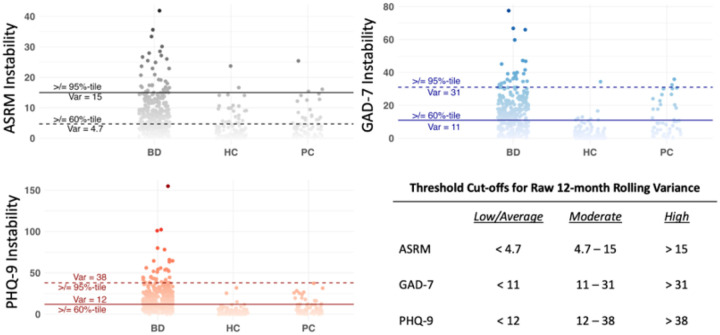
Thresholding of Raw 12-month Rolling Variance Scores Note. Instability = Raw 12-month rolling variance scores. BD = Bipolar Disorders; HC = Healthy Control; PC = Psychiatric Comparison. Solid lines represent the cutoff for moderate instability, dashed lines represent the cutoff for high instability

**Figure 2:**
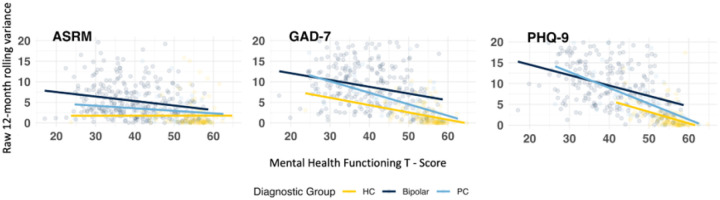
Regressions of 12-month Rolling Variance on Mental Health Functioning T-Scores

Individuals with BD experienced significantly higher instability in ASRM scores than HC (= 0·55, p<.001, large effect) and PC (= 0·41, p<.001, large effect). PC did not differ from HC (= 0·14, p=0·21). Using the 60^th^ and 95^th^ percentile to stratify low, moderate, and high instability, a significantly greater proportion of individuals with BD were above the moderate and high instability thresholds for the ASRM compared to HC and PC and a significantly lower proportion of individuals with BD fell below the low instability threshold (Table 1; Figure 1). These thresholds significantly predicted mental health functioning; those above the high instability threshold for the ASRM had an average T score 1·09 less than those in the low group, holding all other variables constant (Table 2; Figure 2). Interestingly, linear mixed effects models revealed that for the ASRM, physical health functioning improved with higher ASRM instability; those above the moderate instability threshold had a T score of 0·76 higher than those in the low group and those above the high threshold had a T score 1·81 higher than those in the low group, holding all other variables constant (Table 3)

### 3·2 GAD Instability

Instability in GAD-7 scores was significantly greater among the BD group compared to HC (= 0·83, p<.001, large effect) and PC (= 0·37, p<.001, moderate effect). Those in the PC group also had higher instability than HC (= 0·46, p<.001, moderate effect). A significantly greater proportion of individuals with BD were above the moderate and high threshold compared to HC and PC (Table 1; Figure 1). These thresholds significantly predicted mental health functioning; those above the moderate threshold had an average T score of 3·11 less than those in the low group; those above the high instability threshold had an average T score of 3·7 less than those in the low group, holding all other variables constant (Table 2; Figure 2). GAD-7 instability thresholds were not significantly associated with physical health functioning (Table 3)

### 3·3 PHQ Instability

On average, individuals with BD had significantly higher instability in PHQ-9 scores than HC (= 0·79, p<.001, large effect) and PC (= 0·48, p<.001, moderate effect). Those in the PC group did not differ from those in the HC group (= 0·31, p = .03). A signi cantly greater proportion of individuals with BD fell above the moderate and high thresholds compared to HC and PC and a significantly lower proportion fell below the low threshold (Table 1; Figure 1). These thresholds significantly predicted mental health functioning; those above the moderate threshold had an average T score 3·61 less than those in the low group and those above the high threshold had an average T score 5·34 less than those in the low group (Table 2; Figure 2). Instability thresholds for the PHQ-9 were not significantly associated with physical health functioning.

## Discussion

Mood instability is recognized as a core phenotype of BD, yet there are no established methods to measure and index this instability that can be easily and efficiently adapted to clinical trials and/or in the delivery of routine clinical care, such as with PROMs. In a unique longitudinal cohort with deep phenotyping, the PLS-BD, instability was characterized based on the variance of PROMs including the PHQ-9, ASRM, and GAD-7 over rolling 12-month windows. We then identified thresholds that stratified individuals based on low, moderate, and high instability in each mood measure, respectively.

Across all measures, those with BD had significantly higher instability compared to HC and PC with moderate to large effect sizes.The level of instability differed significantly from those in the PC group suggesting that it is not simply a function of psychopathology in general, rather reflective of the trajectory of BD. Only three individuals in the PC group were above the high instability threshold for the ASRM, two above the high instability threshold for the GAD-7, and none were above the high instability threshold for the the PHQ-9. This is suggestive of high specificity of this instability index to the BD group.

Elevated instability in depression was associated with adverse outcomes for those with BD. Moderate and high instability in PHQ-9 scores was significantly associated with both lower mental (large effect) and physical health functioning (small effect). This is consistent with findings from the Global Bipolar Cohort (n = 5,882 individuals with BD) that reported subsyndromal symptoms of depression are among the strongest predictors of poor functioning in BD (6). In contrast, instability in (hypo)manic symptoms was associated with lower mental health (small effect) but better physical health functioning (small effect). However, these small effects are likely clinically insignificant, so further interpretation is cautioned.

Clinically, these results provide guidelines for practical clinical monitoring in the daily patient care setting and offer a novel strategy for outcomes assessments in clinical treatment trials for BD by calculating the variance of PROMs as an index of mood instability. The PHQ-9 and GAD-7 are among the most validated and most widely used PROMs across both primary care and psychiatric settings (22, 26). Given their short length (7 and 9 items), accessibility, and translations into numerous languages, they are easy to integrate into standard operating procedures in primary care and psychiatric settings, research studies, and clinical trials. The ASRM is less widely administered and is used specifically for BD monitoring but reliability of this measure (and most self-report measures of mania) is generally less than that of the PHQ-9 and GAD-7, but remain useful in the longitudinal setting (27). Traditional methods of scoring the PHQ-9, GAD-7, and ASRM focus on a sequential decrease in scores (e.g., PHQ-9 score from 15 to 8 over 12 months) as evidence of a treatment effect. For example, the reliable change index (RCI) in clinical trials calculates the decrease in a score that should be expected based on one’s starting value and regression to the mean (e.g.,(28)). However, in the study of BD it is arguably of greater clinical relevance to monitor mood instability as measured by the variance in common PROMs such as the PHQ-9 scores over a 12 month period. Future investigations must consider that non-linear change (reduction in instability or a shift to a lower instability threshold) may be critical for both understanding the nature, trajectory, and treatment response in BD (8, 9). Once norms for mood instability indices are fully established, future research should investigate the impact that reducing mood instability has on primary outcomes of interest (e.g., physical and mental health functioning, well-being, cognition, interpersonal relationships, occupational outcomes).

Although results provide preliminary thresholds and guidelines for common PROMs that stratify individuals into the low, moderate, and high instability thresholds, next steps include identifying larger samples from electronic health record data or large global research collaboratives such as the Global Bipolar Cohort (GBC; (6, 29)) and National Network for Depression Centers (30) to establish norms based on larger and more diverse samples.

### 4·1 Limitations

A significant limitation of the PLS-BD is its relatively small size and limited ethnic and racial diversity, having been ascertained in a small geographical area. Effect sizes were large for diagnosis; those with BD and PC had significantly lower mental and physical health functioning than HC. While instability is likely intrinsic to the diagnosis, it is di cult to tease out the effect of the BD illness vs. the instability. A further limitation is the limited number of PROM assessments; participants completed PROMs every two months. This was done to minimize participant burden over an extended period of time; however, it is possible that the uctuation of the PROMs is greater than what is picked up by a bi-monthly cadence.

### 4·2 Interpretation

This study outlines a paradigm shift in monitoring outcomes in BD, by measuring mood instability as a primary outcome index. Mood instability, as measured in commonly used PROMs, characterized the course of illness over time, correlated with functional outcomes, and significantly differentiated those with BD from HC and PC. With the growing datasets emerging worldwide, there will be sufficient information from common clinical outcomes measures to assess instability indices of BD globally.

## Figures and Tables

**Table 1.** Instability Thresholds for Each Mood Measure by Diagnosis

	BD	HC	PC	Test statistic
**ASRM Instability Thresholds**	**(N=325)**	**(N=140)**	**(N=64)**	
Low	133 (40.9%)	114 (81.4%)	44 (68.8%)	*X*^2^ (2) = 45.30, *p*<.001
Medium	157 (48.3%)	24 (17.1%)	17 (26.6%)	*X*^2^ (2)= 188.58, *p*<.001
High	35 (10.8%)	2 (1.4%)	3 (4.7%)	*X*^2^ (2)= 52.85, *p*<.001
**GAD-7 Instability Thresholds**	**(N=286)**	**(N=132)**	**(N=55)**	
Low	113 (39.5%)	126 (95.5%)	39 (70.9%)	*X*^2^ (2)= 47.53, *p*<.001
Medium	147 (51.4%)	5 (3.8%)	14 (25.5%)	*X*^2^ (2)= 228.52, *p*<.001
High	26 (9.1%)	1 (0.8%)	2 (3.6%)	*X*^2^ (2)= 41.45, *p*<.001
**PHQ-9 Instability Thresholds**	**(N=322)**	**(N=140)**	**(N=66)**	
Low	115 (35.7%)	136 (97.1%)	49 (74.2%)	X^2^ (2)= 41.22, p<.001
Medium	170 (52.8%)	4 (2.9%)	17 (25.8%)	*X*^2^ (2)= 267.72, *p*<.001
High	37 (11.5%)	0 (0%)	0 (0%)	*X*^2^ (2)= 74, *p*<.001

**Note**. BD = bipolar disorders; HC = health controls; PC = psychiatric comparison. Note that sample sizes differ by measure given different levels of missingness exist for each measure.

**Table 2.** Instability Thresholds Predicting Longitudinal Mental Health Functioning

	ASRM predicting MCS			GAD-7 Predicting MCS			PHQ-9 Predicting MCS		
*Predictor*	*Estimates*	*Conf. Int (95%)*	*P-Value*	*Estimates*	*Conf. Int (95%)*	*P-Value*	*Estimates*	*Conf. Int (95%)*	*P-Value*
(Intercept)	51.71	49.19 – 54.24	**<0.001**	50.74	48.03 – 53.45	**<0.001**	51.99	49.62 – 54.37	**<0.001**
Medium vs. Low/A	−**0.51**	−0.81 − −0.20	**0.001**	−**3.11**	−3.49 - −2.74	**<0.001**	−**3.61**	−3.93 - −3.30	**<0.001**
High vs. Low/A	−**1.09**	−1.67 - −0.51	**<0.001**	−**3.7**	−4.40 - −3.00	**<0.001**	−**5.34**	−5.94 - −4.74	**<0.001**
BD v HC	−**14.12**	−15.72 - −12.53	**<0.001**	−**12.61**	−14.27 - −10.94	**<0.001**	−**12.32**	−13.82 - −10.82	**<0.001**
PC v HC	−**5.96**	−8.27 - −3.65	**<0.001**	−**6.4**	−8.86 - −3.94	**<0.001**	−**5.33**	−7.49 - −3.17	**<0.001**
BD v PC	−**8.12**	−10.49 - −5.75	**<0.001**	−**6.27**	−8.81 - −3.74	0.704	−**7.01**	−9.24 - −4.79	**<0.001**
Is Female	−0.78	−2.23 – 0.66	0.288	−0.31	−1.88 – 1.27	0.691	−0.43	−1.79 – 0.93	0.536
Is Non-White	0.68	−1.05 – 2.40	0.442	0.37	−1.46 – 2.20	0.001	0.43	−1.19 – 2.05	0.601
Age at Enrollment	0.08	0.03 – 0.13	0.002	**0.09**	0.04 – 0.14		0.07	0.03 – 0.12	0.002

**Note**. MCS = SF-12 Mental Health Functioning T Score (M = 50, SD = 10). Estimates are unstandardized betas for interpretation of +/− T score values. Medium vs. Low/A = Medium vs. Low/Average Threshold; High vs. Low/A = High vs. Low/Average Threshold. BD = bipolar disorders; HC = healthy control; PC = psychiatric comparison. Significant associations with alpha <= .001 are bolded.

**Table 3.** Instability Thresholds Predicting Longitudinal Physical Health Functioning

	ASRM predicting PCS			GAD-7 Predicting PCS			PHQ-9 Predicting PCS		
*Predictor*	*Estimates*	*Conf. Int (95%)*	*P-Value*	*Estimates*	*Conf. Int (95%)*	*P-Value*	*Estimates*	*Conf. Int (95%)*	*P-Value*
(Intercept)	60.93	58.31 – 63.55	**<0.001**	60.09	57.14 – 63.05	**<0.001**	61.15	58.55 – 63.75	**<0.001**
Medium vs. Low/A	**0.76**	0.53 – 1.00	**<0.001**	0.33	0.05 – 0.62	0.023	−0.35	−0.60 - −0.11	0.005
High vs. Low/A	**1.81**	1.36 – 2.26	**<0.001**	0.39	−0.14 – 0.92	0.152	0.04	−0.43 – 0.51	0.876
BD v HC	−**6.8**	−8.46 − −5.15	**<0.001**	−**6.63**	−8.44 - −4.83	**<0.001**	−**6.27**	−7.92 - −4.63	**<0.001**
PC v HC	−3.18	−5.57 - −0.78	0.009	−3.57	−6.25 - −0.90	0.009	−3.01	−5.37 - −0.64	0.013
BD v PC	−3.47	−5.96 - −0.98	0.006	−2.93	−5.71 - −0.14	0.04	−3.16	−5.62 - −0.69	0.012
Is Female	−1.46	−2.96 – 0.04	0.056	−1.14	−2.86 – 0.57	0.192	−1.42	−2.91 – 0.08	0.063
Is Non-White	−0.97	−2.76 – 0.82	0.288	−0.46	−2.45 – 1.52	0.647	−0.87	−2.64 – 0.90	0.335
Age at Enrollment	−**0.2**	−0.25 - −0.15	**<0.001**	−0.2	−0.26 - −0.14	**<0.001**	−**0.21**	−0.26 - −0.16	**<0.001**

**Note**. PCS = SF-12 Mental Health Functioning T Score (M = 50, SD = 10). Estimates are unstandardized betas for interpretation of +/− T score values. Medium vs. Low/A = Medium vs. Low/Average Threshold; High vs. Low/A = High vs. Low/Average Threshold. BD = bipolar disorders; HC = healthy control; PC = psychiatric comparison. Signicant associations with alpha <= .001 are bolded.

## Data Availability

Longitudinal and outcomes data used in the present study, along with data dictionaries, are available subject to review of the proposed analyses and acceptance of a Data Use Agreement. All PLS-BD data and samples are available through the Heinz C. Prechter Genetic Repository, distributed by the University of Michigan Central Biorepository (CBR). Enquiries can be addressed at http://www.prechterprogram.org/data.

## Footnotes

[1] Note that much of the literature refers to this construct as emotion or mood “instability.” While we agree conceptually, statistically, the majority of these studies focus on variability, or, the intraindividual standard deviation or variance in responses rather than the mean square of successive differences (MSSD), the statistical measure of instability. See Sperry & Kwapil 2022 for a longer discussion of this issue.

[2] Within-person observations for the GAD-7 were significantly lower because the GAD-7 did not begin to be administered until year 5 of the study causing much lower number of measured instances.
